# Analysis of the Performance and Sailing Variables of the Optimist Class in a Variety of Wind Conditions

**DOI:** 10.3390/jfmk9010018

**Published:** 2024-01-03

**Authors:** Israel Caraballo, Luka Pezelj, Juan José Ramos-Álvarez

**Affiliations:** 1 GALENO Research Group, Department of Physical Education, Faculty of Education Sciences, University of Cádiz, 11519 Puerto Real, Spain; 2 Instituto de Investigación e Innovación Biomédica de Cádiz (INiBICA), 11009 Cádiz, Spain; 3 Faculty of Maritime Studies, University of Split, 21000 Split, Croatia; lpezelj@pfst.hr; 4 Escuela de Medicina Deportiva, Departamento de Radiología, Rehabilitación y Fisioterapia, Universidad Complutense de Madrid, 28040 Madrid, Spain; jjramosa@med.ucm.es

**Keywords:** GPS, sailors, sport performance, tactics, elite, GNSS

## Abstract

The aim of this study was to analyse the variables that determine the performance of the Optimist class during a regatta in different wind conditions. A total of 203 elite sailors of the Optimist class (121 boys and 82 girls) participated in the study. According to their ranking in the regatta, the sample was divided into four performance groups. In a regatta with 11 races, the velocity made good (VMG), the distance and the manoeuvres were evaluated by means of GNSS equipment in three different courses. The boys performed a greater number of upwind and running manoeuvres than the girls. The very-low-level sailors obtained a lower VMG in all the courses analysed compared with the rest of the groups of sailors of higher levels. Upwind manoeuvres, broad reach and running VMG were significant variables for establishing differences in performance level when the wind speed was in a range of 5 to ≤8 knots. When the wind speed was in the >8 to ≤12 knot range, upwind distance was the key variable in determining performance differences. VMG, upwind and broad reach distance and broad reach manoeuvres were the most important variables when the wind speed was in the >12 to 15 knots range. The boys performed more manoeuvres than the girls in the upwind and running courses.

## 1. Introduction

Among the different dinghy sailing classes, the Optimist class is part of the monohull category and is crewed by a single sailor. This class is governed by the class rules and uses a boat with a weight of 35 kg, a length of 2.36 m, a beam of 1.12 m and a main sail area of 3.32 m^2^. In the Optimist class, boys and girls up to the age of 15 years compete together [1]. This class has been proposed by the International Sailing Federation and has its own organisation at the international level: The International Optimist Dinghy Association. In the course racing discipline, the sailors must complete a race in the shortest time possible, sailing upwind, on a broad reach and running in a course marked by buoys.

Dinghy sailing is a multifaceted sport where performance is determined by numerous factors, such as morphology, psychological and physical fitness, and technical and tactical skills [2,3]. Moreover, performance is also influenced by the characteristics of the boat and the weather conditions [4,5]. The factors that determine performance in a regatta are technique (speed) and tactics (distance and manoeuvres) [6,7]. The most important variable in a regatta is the speed of the boat and the velocity made good (VMG) on the windward and leeward courses [8]. Other studies have shown that elite sailors complete the course using a shorter distance [9,10]. Where manoeuvres are concerned, studies have shown that the most successful sailors perform fewer upwind manoeuvres [11,12].

Regarding performance, several studies have analysed the relationship between performance and technical and tactical skills in the windsurfing [6,11], Laser class [8,13], 2.4mR class [7] and Formula Kite class [14,15]. However, to our knowledge, not all aspects of technical and tactical performance have been thoroughly examined in the Optimist class. Therefore, the aim of our study was to identify the variables that determine sport performance in Optimist class sailors under different wind conditions.

## 2. Materials and Methods

### 2.1. Participants

The study sample consisted of 203 international elite sailors (82 girls) of the Optimist class, with an age range of 9 to 15 years, who competed in an international regatta. The data were collected from World-Sailing, and they were obtained from a publicly accessible website [16]. Thus, ethical approval and written/informed consent from all participants were not necessary. To perform the analysis based on wind speed, the total sample of 203 sailors was divided into four performance groups according to the ranking of the sailors in the regatta: high-level sailors (P_25_), medium-level sailors (P_50_), low-level sailors (P_75_) and very-low-level sailors (P_99_). The percentile values in the ranking (25th, 50th and 75th) were used to divide the sample into P_25_ (*n* = 51; 18 girls), P_50_ (*n* = 51; 22 girls), P_75_ (*n* = 51; 16 girls) and P_99_ (*n* = 50; 26 girls).

### 2.2. Regatta

**Figure 1:**
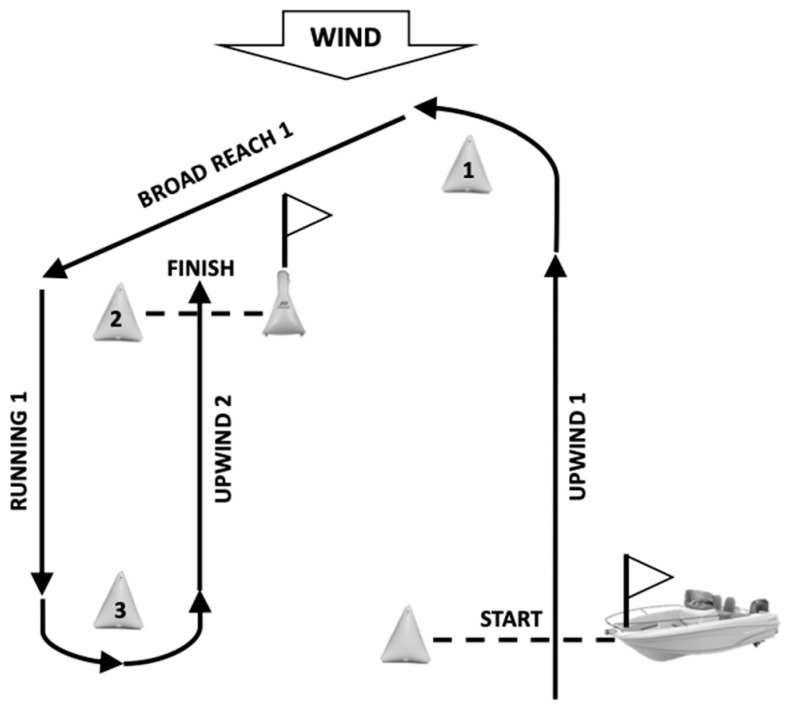
Regatta race course.

The analysed regatta was the Travemünder Woche 2017. This regatta was an international competition and it was a qualifying competition for the World Cup, although only the results obtained in the ranking of this regatta were used. The average values of the velocity made good (knots), distance (km) and manoeuvres (number of manoeuvres) variables during the upwind, broad reach and running courses were obtained through a SAP-Sailing application [17]. This application uses a global navigation satellite system device (GNSS) placed on the sailor’s boat. From this device, data are transmitted and processed in real time by the application, obtaining information about those variables. The average values of variables during each course were obtained from the regatta and the races. Furthermore, wind speed was analysed in all races of the regatta. The ranking in the regatta and a single race was used to determine the performance of the sailors, which allowed classifying the athletes in the different performance groups. A total of 11 races were analysed. The race course consisted of four legs: two upwind, one broad reach and one running (Figure 1). The wind speed in the regatta ranged between 2.8 and 16.4 knots. Wind speed was categorised in each race according to the Royal Yachting Association [18]: 5 to ≤8 knots (light wind), >8 to ≤12 knots (medium wind) and >12 to 15 knots (strong wind). Wind speed was measured continuously and the maximum and minimum values were used to calculate the average for each race.

### 2.3. Statistical Analysis

The data are presented as mean and standard deviations (SD). The level of significance was set at *p* < 0.05. SPSS v20.0 software (SPSS Lead Technologies Inc., Chicago, IL, USA) was used for the statistical analyses. The data were subjected to a descriptive analysis and inferences. The normality of all variables was verified using the Kolmogorov–Smirnov test and the Levene´s test was used to evaluate the homogeneity of variance. ANOVA was used to determine the possible differences, based on sex (males and females) and performance level (P_25_, P_50_, P_75_ and P_99_). Under the assumption of equal variances, a Bonferroni post hoc test was used when statistically significant differences were detected and a Games–Howell post hoc was applied to establish differences for variables with unequal variances. The effect size was calculated using partial eta-squared ($\eta_{p}^{2}$), considering <0.25, 0.26–0.63, and >0.63 as small, medium, and large effect size, respectively [19,20]. The ranges in wind speed in each of the races were analysed to study the effect of the interaction between the performance level and wind speed on performance.

## 3. Results

Table 1 shows the descriptive statistics of the variables for the total sample and by sex in the regatta. It was observed that the boys performed a larger number of manoeuvres in the upwind and running courses compared to the girls. No statistically significant differences were observed in the variables age, upwind VMG, upwind distance, broad reach VMG, broad reach distance, broad reach manoeuvres, running VMG, running distance, or ranking.

**Figure 2:**
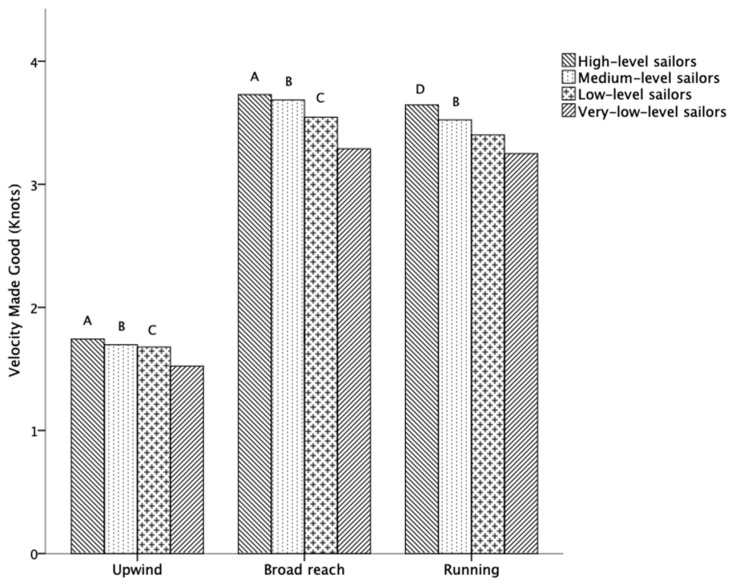
Comparison between the groups of sailors with different performance levels in upwind, broad reach and running courses for velocity made good. Note: **A**: statistically significant difference between high-level and very-low-level sailors; **B**: statistically significant difference between medium-level and very-low-level sailors; **C**: statistically significant difference between low-level and very-low-level sailors; **D**: statistically significant difference between high-level and low-level and very-low-level sailors; statistical significance level: *p* < 0.05.

Figure 2 shows the analysis for each of the groups of sailors according to their level of performance in the regatta. It was observed that, in the upwind (1.7 ± 0.2 vs. 1.5 ± 0.2 knots; *p* < 0.01), broad reach (3.7 ± 0.4 vs. 3.2 ± 0.4 knots; *p* < 0.01) and running (3.6 ± 0.3 vs. 3.2 ± 0.4 knots; *p* < 0.01) courses, the high-level sailors obtained greater VMG compared to the very-low-level sailors. Medium-level sailors had a greater VMG compared to the very-low-level sailors in the upwind (1.6 ± 0.1 vs. 1.5 ± 0.2 knots; *p* < 0.05), broad reach (3.6 ± 0.3 vs. 3.2 ± 0.4 knots; *p* < 0.01) and running (3.5 ± 0.3 vs. 3.2 ± 0.4 knots; *p* < 0.01). Similarly, the VMG was higher in the low-level sailors compared to the very-low-level group in the upwind (1.6 ± 0.2 vs. 1.5 ± 0.2 knots; *p* < 0.01) and broad reach (3.54 ± 0.4 vs. 3.2 ± 0.4 knots; *p* < 0.05). With regard to the broad reach distance in the regatta, the very-low-level sailors travelled a shorter distance compared to the high-level sailors (0.9 ± 0.1 vs. 0.8 ± 0.1 km; *p* < 0.05). No statistically significant differences were observed in the other variables.

When the races were assessed, the results showed that the high-level sailors had a greater VMG in the broad reach (3.3 ± 0.9 vs. 2.9 ± 1.3 knots; *p* < 0.01) and running (3.1 ± 0.8 vs. 2.7 ± 1.2 knots; *p* < 0.01) courses compared to the very-low-level sailors in a light wind (Table 2). Regarding strong wind conditions, the high-level sailors presented a higher value of upwind VMG compared to the medium-level (1.9 ± 0.3 vs. 1.8 ± 0.3 knots; *p* < 0.05), low-level (1.9 ± 0.3 vs. 1.7 ± 0.4 knots; *p* < 0.01) and very-low-level (1.9 ± 0.3 vs. 1.6 ± 0.4 knots; *p* < 0.01) sailors. In all the wind conditions analysed, higher VMG values were achieved. When analysing the distances travelled in the different wind speed conditions, it was observed that the high-level sailors travelled a shorter upwind distance than the very-low-level (1.3 ± 0.3 vs. 1.4 ± 0.3 km; *p* < 0.05) sailors in a medium wind. In strong wind conditions, the results showed that the upwind distance was shorter in the high-level sailors (1.3 ± 0.2 vs. 1.4 ± 0.3 km; *p* < 0.05) compared to the very-low-level sailors. However, the high-level sailors travelled a longer broad reach distance in strong wind conditions compared to the low-level (1.1 ± 0.2 vs. 1 ± 0.2 km; *p* < 0.01) and very-low-level sailors (1.1 ± 0.2 vs. 1 ± 0.2 km; *p* < 0.01). With respect to the number of manoeuvres, compared to the very-low-level sailors, the high-level sailors presented a larger number of upwind manoeuvres in a light wind (14.3 ± 7.6 vs. 12.2 ± 9.1 number; *p* < 0.05). Nevertheless, when the number of manoeuvres was analysed in strong wind conditions, it was observed that the high-level sailors had a greater value of broad reach manoeuvres compared to the medium-level (0.6 ± 0.8 vs. 0.3 ± 0.7 manoeuvres; *p* < 0.01) and low-level (0.6 ± 0.8 vs. 0.3 ± 0.8 manoeuvres; *p* < 0.01) sailors.

## 4. Discussion

The aim of this study was to identify the variables that determine a sailor’s performance in different wind conditions.

The results of our study showed that the boys performed a larger number of upwind manoeuvres and running manoeuvres in comparison to the girls. VMG is the variable that differentiates sailors according to their level of performance on the three courses analysed. In terms of wind speed, statistical differences in upwind manoeuvres, broad reach VMG and running VMG were reported between the high-level and low-level sailors in light wind conditions (5 to ≤8 knots). In medium wind conditions (>8 to ≤12 knots), it was observed that the high-level sailors covered a shorter upwind distance than the very-low- level sailors. When the wind was strong (>12 to15 knots), the results showed that the high-level sailors achieved a higher VMG on all courses, travelled a shorter distance upwind, covered a shorter distance in broad reach, and performed fewer broad reach manoeuvres.

Regarding sex, statistically significant differences were only found in the number of manoeuvres in upwind and running, with the boys performing more manoeuvres than the girls. Although it has been observed that the larger the number of manoeuvres that were performed, the greater was the effort required by the sailor [12], with this action reducing the velocity of the boat [21], the larger the number of manoeuvres that were performed, the better was the orientation of the boat to reach the buoy [13,22]. This could explain the larger number of manoeuvres in the upwind and running courses performed by the boys compared to the girls. In this regatta, both boys and girls sailed the same course; thus, they were exposed to the same wind speed and wave conditions. Therefore, these results could be relevant to coaches when planning the training of sailors based on sex. To the best of our knowledge, this is the first study to compare the technical and tactical variables of elite Optimist sailors by sex during a regatta.

The data obtained from our sailors in the regatta showed that the high-level sailors achieved the highest VMG in each of the courses (upwind, broad reach and running) compared to the very-low-level sailors. Similarly, the higher-level groups of sailors sailed faster (VMG) in the upwind, broad reach and running courses compared to the lower-level sailors. VMG as a performance factor in the courses of upwind, broad reach and running, in terms of average VMG, has also been confirmed in other classes, such as Formula Kite, Windsurfing and Laser [8,9,10,14]. This could suggest that the most successful group of sailors have a better technical level, which would allow them to reach higher speeds in each of the courses [23]. Therefore, and based on our results, we would assert that, in our Optimist class sailors, the higher the level of the sailor is, the greater is the VMG achieved in the upwind, broad reach and running courses. The results of our study could be very interesting for coaches and sailors, since they indicate that the VMG of the elite sailor is not affected by a specific course. Thus, improving the VMG in each of the courses might be the target of the training sessions.

Analysing the wind speed in each of the races, it was found that the variables that determine performance in dinghy sailing would be different depending on the wind speed in the regatta. In light winds (5 to ≤8 knots), our results suggest that upwind manoeuvres, broad reach VMG and running VMG may be the variables that determine sailor performance. The high-level and medium-level sailors had a higher VMG than the very-low-level sailors. This may also mean that the most successful sailors handle the boat more efficiently [24]. With regard to the number of manoeuvres, in the Laser class, it has been observed that the number of manoeuvres performed by the sailor in upwind increases in light wind conditions [22]. In these wind conditions, the sailor must make a larger number of tacks to find the most favourable wind zones that will allow him/her to advance until he/she reaches the windward buoy. Moreover, it has been shown that the angle between the wind and the bow must be approximately 40° in order to reduce the distance travelled by the board against the wind [24]. In medium wind conditions (>8 to ≤12 knots), it was observed that the most successful sailors sailed a shorter distance upwind compared to the less successful sailors. Our results are in line with those of sailors in the windsurfing class [9,10]. Our data showed no statistically significant differences in VMG or manoeuvres. We can thereby confirm that, in these wind conditions, it is the upwind distance which determines performance in the Optimist class. The most successful sailors would use better tactics to complete the course in a shorter distance [8,25]. Therefore, our results suggest that training for the Optimist class could be focused on improving upwind distance when the wind speed is between >8 and ≤12 knots. When the wind speed was between >12 and 15 knots, our results showed that the most successful sailors had a higher VMG in all courses, although it should be noted that the high-level sailors had a higher upwind speed compared to the other three groups. Previous studies have also shown that VMG is considered an important variable in regattas, with more successful sailors having a higher VMG in both upwind and running legs [26]. Thus, our findings suggest that VMG in the upwind course could be a variable that determines the performance of the sailor in the Optimist class in strong wind speeds. As with >8 to ≤12 knot winds, the high-level sailors covered a shorter distance upwind, although they covered a longer distance in broad reach compared to the low-level sailors. In a similar way to the windsurfing class, it can be assumed that the longer distance travelled by the top sailors is compensated for by their higher speed, which allows them to reach their destination in a shorter time [11]. In broad reach, to achieve a better planning of the boat and thus a higher speed, the sailor can change the orientation of the boat in relation to the wind, although this increased angle may also increase the distance travelled. Regarding manoeuvres, it was observed that the high-level sailors performed a greater number of manoeuvres in broad reach compared to the rest of the sailors. In Laser class sailors, it has been observed that the number of downwind manoeuvres increased with the increase in wind speed [13]. Our results could be explained by the fact that, in strong winds, the technical action of hiking puts pressure on the abdominal and quadriceps muscles, restricting blood flow and increasing muscle fatigue. When the sailor performs manoeuvres, he/she changes position, reducing the pressure on the muscles and increasing blood flow [27,28]. This reduces muscle fatigue by increasing oxygen consumption in the abdominal and quadriceps muscles. Therefore, and considering that the manoeuvres reduce the speed of the boat and can also lead to greater fatigue in the sailor [11,22], the latter must train to increase efficiency in the manoeuvres and thus reduce the loss of speed of the boat in strong wind conditions [24]. Our results are consistent with those obtained by Chun et al. [6], and we can therefore confirm that the technical and tactical variables played an important role when starting in strong wind conditions.

## 5. Strengths and Limitations

Our study is not exempt from limitations. Firstly, the anthropometric characteristics of the sailors were not collected. It is possible that the performance of the sailor may be influenced by the variables in weight and height. Unfortunately, it was not possible to include such data in this study, although it would have been interesting to analyse these variables, which could provide additional information to better explain results. Secondly, it would be interesting to assess some of the relevant aspects involved in the physical performance and physiological demands of Optimist course racing. In addition, future studies could focus on analysing these variables in different wind conditions and specifically analyse each of the races that make up the regatta.

To our knowledge, this is the first study to evaluate VMG, distance and manoeuvres in a real regatta situation as a function of the performance of the sailors, specifically in Optimist class sailors, and it is also the first study to analyse the three types of courses developed during a regatta (upwind, broad reach and running).

## 6. Conclusions

In the Optimist class, sailor performance is determined by upwind, broad reach and running VMG in all types of wind speed conditions. Performance in the Optimist class is determined by the distance travelled upwind in winds between 8 and 12 knots.

## Figures and Tables

**Table 1:** Mean ± SD of the variables analysed in all sailors and in girls and boys.

Variable	Total Sample (*n* = 203)	Girls (*n* = 82)	Boys (*n* = 121)	ANOVA	
				*p*-Value	$E_{R}^{2}$
Age (years)	13.1 ± 1.2	13.2 ± 1	13 ± 1.3	0.229	0.00
Upwind VMG (knots)	1.6 ± 0.3	1.6 ± 0.2	1.6 ± 0.3	0.334	0.04
Upwind distance (km)	1.2 ± 0.1	1.2 ± 0.1	1.3 ± 0.1	0.942	0.00
Upwind manoeuvres (number)	9.3 ± 2.1	8.7 ± 2.1	9.5 ± 1.9 *	0.005	0.03
Broad reach VMG (knots)	3.5 ± 0.4	3.5 ± 0.4	3.5 ± 0.4	0.194	0.01
Broad reach distance (km)	0.9 ± 0.1	0.9 ± 0.1	0.9 ± 0.1	0.690	0.00
Broad reach manoeuvres (number)	0.4 ± 0.3	0.4 ± 0.4	0.4 ± 0.3	0.962	0.00
Running VMG (knots)	3.4 ± 0.4	3.4 ± 0.4	3.4 ± 0.3	0.157	0.01
Running distance (km)	0.8 ± 0.1	0.7 ± 0.1	0.8 ± 0.1	0.103	0.01
Running manoeuvres (number)	3.5 ± 1	3.3 ± 1.1	3.6 ± 0.1 *	0.022	0.02
Ranking	102 ± 58.7	107.8 ± 60.2	98.1 ± 57.6	0.247	0.00

Note: VMG: Velocity Made Good; $E_{R}^{2}$ = effect size. *: Statistically significant difference between boys and girls (*p* < 0.05).

**Table 2:** Mean ± SD of the variables analysed in the percentile groups according to wind speed in races.

Wind Speed	Variable	P_25_ (*n* = 51)	P_50_ (*n* = 51)	P_75_ (*n* = 51)	P_99_ (*n* = 50)	ANOVA	
						*p*-Value	$E_{R}^{2}$
	Upwind VMG (knots)	1.6 ± 0.5	1.6 ± 0.4	1.6 ± 2.5	1.3 ± 0.6	0.083	0.01
	Upwind distance (km)	1 ± 0.3	1 ± 0.3	1.1 ± 0.4	1.1 ± 0.4	0.190	0.00
	Upwind manoeuvres (number)	14.3 ± 7.6 ^E^	14 ± 7.4	12.8 ± 7.9	12.2 ± 9.1	0.021	0.01
	Broad reach VMG (knots)	3.3 ± 0.9 ^E^	3.2 ± 0.8 ^G^	3.2 ± 1.1	2.9 ± 1.3	0.001	0.02
5 to ≤8	Broad reach distance (km)	0.8 ± 0.2	0.8 ± 0.2 ^F^	0.8 ± 0.3	0.7 ± 0.3	0.011	0.01
(knots)	Broad reach manoeuvres (number)	0.4 ± 0.9	0.5 ± 1.9	0.5 ± 1.5	0.5 ± 2	0.697	0.00
	Running VMG (knots)	3.1 ± 0.8 ^E^	3 ± 0.8 ^G^	2.9 ± 1	2.7 ± 1.2	0.000	0.02
	Running distance (km)	0.6 ± 0.3	0.6 ± 0.3	0.5 ± 0.3	0.5 ± 0.3	0.069	0.01
	Running manoeuvres (number)	2.7 ± 2.8	2.7 ± 3	2.5 ± 3.1	2.6 ± 3.1	0.857	0.00
	Upwind VMG (knots)	1.6 ± 0.4	1.6 ± 0.3	1.6 ± 0.3	1.5 ± 0.4	0.297	0.00
	Upwind distance (km)	1.3 ± 0.3 ^E^	1.4 ± 0.3	1.4 ± 0.2	1.4 ± 0.3	0.027	0.01
	Upwind manoeuvres (number)	21 ± 8.1	22 ± 8.1	21.2 ± 8.9	21.2 ± 9	0.620	0.00
	Broad reach VMG (knots)	3.8 ± 0.9	3.8 ±0.9	3.8 ± 0.8	3.7 ± 1	0.470	0.00
>8 to ≤12	Broad reach distance (km)	0.8 ± 0.2	0.8 ± 0.1	0.8 ± 0.1	0.8 ± 0.2	0.764	0.00
(knots)	Broad reach manoeuvres (number)	0.2 ± 0.5	0.1 ± 0.4	0.2 ± 0.7	0.3 ± 1.7	0.270	0.00
	Running VMG (knots)	3.4 ± 0.9	3.4 ± 0.8	3.5 ± 0.7	3.3 ± 0.9	0.588	0.00
	Running distance (km)	0.8 ± 0.2	0.8 ± 0.2	0.9 ± 0.1	0.8 ± 0.2	0.561	0.00
	Running manoeuvres (number)	4.4 ± 3.3	4.1 ± 2.7 ^F^	5.1 ± 3.1	4.7 ± 3.1	0.029	0.01
	Upwind VMG (knots)	1.9 ± 0.3 ^A^	1.8 ± 0.3 ^G^	1.7 ± 0.4	1.6 ± 0.4	0.000	0.02
	Upwind distance (km)	1.3 ± 0.2 ^E^	1.4 ± 0.2	1.4 ± 0.3	1.4 ± 0.3	0.021	0.01
	Upwind manoeuvres (number)	20.8 ± 7.7	20.7 ± 8.1	21.2 ± 7.9	22.3 ± 8	0.178	0.00
>12 to 15	Broad reach VMG (knots)	4 ± 0.7 ^C^	3.9 ± 0.8 ^H^	3.6 ± 1.1 ^I^	3.3 ± 1.2	0.000	0.07
(knots)	Broad reach distance (km)	1.1 ± 0.2 ^C^	1 ± 0.2	1 ± 0.2	1 ± 0.2	0.00	0.02
	Broad reach manoeuvres (number)	0.6 ± 0.8 ^B^	0.3 ± 0.7	0.3 ± 0.8	0.5 ± 1.1	0.01	0.01
	Running VMG (knots)	4 ± 0.7 ^C^	3.9 ± 0.8 ^H^	3.9 ± 0.9	3.6 ± 1.2	0.00	0.06
	Running distance (km)	0.9 ± 0.1	0.9 ± 0.2	0.9 ± 0.2	0.9 ± 0.2	0.170	0.00
	Running manoeuvres (number)	3.3 ± 2.2	3.7 ± 2.4	3.6 ± 2.7	3.5 ± 2.5	0.369	0.00

Note: VMG: Velocity Made Good: ^A^: statistically significant difference between P_25_ vs. P_50_, P_75_ and P_99_; ^B^: statistically significant difference between P_25_ vs. P_50_ and P_75_; ^C^ statistically significant difference between P_25_ vs. P_75_ and P_99_; ^E^: statistically significant difference between P_25_ and P_99_; ^F^: statistically significant difference between P_50_ and P_75_; ^G^: statistically significant difference between P_50_ and P_99_; ^H^: statistically significant difference between P_50_ vs. P_75_ and P_90_; ^I^: statistically significant difference between P_75_ and P_99_; P_25_: high-level sailors; P_50_: medium-level sailors; P_75_: low-level sailors; P_99_: very-low-level sailors; $E_{R}^{2}$ = effect size; statistical significance level: *p* < 0.05.

## Data Availability

Publicly available datasets were analysed in this study. These data can be found in https://www.sapsailing.com/gwt/Home.html#EventsPlace: (accessed on 12 February 2021).
